# Diagnostic performance of a fully automated AI algorithm for lesion detection and PI-RADS classification in patients with suspected prostate cancer

**DOI:** 10.1007/s11547-025-02003-0

**Published:** 2025-04-17

**Authors:** Hannes Engel, Andrea Nedelcu, Robert Grimm, Heinrich von Busch, August Sigle, Tobias Krauss, Christopher L. Schlett, Jakob Weiss, Matthias Benndorf, Benedict Oerther

**Affiliations:** 1 Department of Radiology, Medical Center - University of Freiburg, Faculty of Medicine, University of Freiburg, Freiburg, Germany; 2 Siemens Healthineers AG, Forchheim, Germany; 3 Department of Urology, Medical Center - University of Freiburg, Faculty of Medicine, University of Freiburg, Freiburg, Germany; 4 Berta-Ottenstein-Programme, Faculty of Medicine, University of Freiburg, Freiburg, Germany; 5 Department of Diagnostic and Interventional Radiology, Medical Faculty OWL, University of Bielefeld, Klinikum Lippe, Detmold, Germany

**Keywords:** Prostate cancer, Prostatic neoplasms, Multiparametric MRI, PI-RADS, Artificial intelligence

## Abstract

**Purpose:**

To evaluate the diagnostic performance of a fully automated, commercially available AI algorithm for detecting prostate cancer and classifying lesions according to PI-RADS.

**Material and methods:**

In this retrospective single-center cohort study, we included consecutive patients with suspected prostate cancer who underwent 3T MRI between May 2017 and May 2020. Histopathological ground truth was targeted transperineal ultrasound-fusion guided biopsy and extensive systematic biopsy. We compared the results of the AI algorithm to those of human readers on both the lesion and patient level and determined the diagnostic performance.

**Results:**

A total of 272 patients with 436 target lesions were evaluated. Of these patients, 135 (49.6%) had clinically significant prostate cancer (sPCa), 35 (12.9%) had clinically insignificant prostate cancer (ISUP = 1), and 102 (37.5%) were benign. On patient level, the cancer detection rates of sPCa for AI versus human readers were 11% versus 18% for PI-RADS ≤ 2, 27% versus 11% for PI-RADS 3, 54% versus 41% for PI-RADS 4, and 74% versus 92% for PI-RADS 5. The AI showed significantly higher accuracy: 74% versus 63% for PI-RADS ≥ 4 (*p* < 0.01) and 70% versus 52% for PI-RADS ≥ 3 (*p* < 0.01). Additionally, the AI correctly classified 62 patients with human reading PI-RADS ≥ 3 as true negatives.

**Conclusion:**

The AI algorithm proved to be a reliable and robust tool for lesion detection and classification. Its cancer detection rates and PI-RADS category distribution align with the results of recent meta-analyses, indicating precise risk stratification.

**Supplementary Information:**

The online version contains supplementary material available at 10.1007/s11547-025-02003-0.

## Introduction

Multiparametric MRI (mpMRI) represents the imaging tool of choice in the diagnostic pathway of prostate carcinoma (PCa) and is hence incorporated in current international guidelines [1, 2]. Indications include suspicion of PCa without prior biopsy, persisting suspicion after negative biopsy, and risk stratification prior to inclusion into active surveillance. Demand for examinations rises rapidly, further aggravating the problem of already scarce scan capacities [3–5]. Computer-aided detection aims to enhance and accelerate human reading and reassure the reader [6–8]. If thoroughly validated, this approach may enable a much-needed increase in reading capacities. Future diagnostic pathways may be refined by AI-driven radiologic-pathologic integration, reducing interreader variability while optimizing lesion detection, classification, and risk stratification [9].

Suspicious lesions in mpMRI are classified according to the Prostate Imaging Reporting and Data System lexicon (PI-RADS) [10]. Scores correlate with the probability of cancer and translate into recommendations for clinical management, particularly image-guided biopsy.

Current AI-based software solutions for automated detection and classification of prostate lesions appear promising, but most of them are not yet suitable for clinical practice due to a lack of generalizability. Algorithms differ in methodology and are not sufficiently comparable as they are trained using different types and sizes of data sets with different ground truth labels, underscoring the need for thorough validation in large heterogeneous clinical cohorts. [11]

The aim of this study was to externally validate the diagnostic accuracy of a commercially available AI-based algorithm for detection and classification of PCa compared to human readers in a large tertiary referral center.

## Materials and methods

### Subjects

In this retrospective single-center cohort study we evaluated 310 consecutive patients without known PCa with mpMRI of the prostate performed between 05/2017 and 05/2020, followed by targeted transperineal ultrasound-fusion guided and extensive systematic biopsy. 38 patients were excluded due to missing clinical data or histopathological confirmation, resulting in 272 patients. The institutional ethics committee approved the study and waived informed consent (approval number: 20–1256). Indications for MRI were suspicious (prostate specific antigen) PSA (> 4 ng/ml or increase of 0.3–0.7 ng/ml/year) or abnormal digital rectal examination.

### MRI protocol and reading

The examinations were performed in a 3T MRI scanner (MAGNETOM VIDA, Siemens Healthineers, Erlangen, Germany) according to the PI-RADSv2.1 protocol. Scan parameters were as follows: T2-weighted axial images (2D TSE; slice thickness: 3 mm; no gap; TR: 7500 ms; TE: 104 ms, flip angle: 160 °; field-of-view: 200 × 200 mm; 768 × 768 matrix; in-plane resolution 0.26 × 0.26 mm), axial diffusion-weighted sequence (RESOLVE; slice thickness: 3 mm; no gap; TR: 4300 ms; TE: 74 ms; flip angle: 90 °; field-of-view: 200 × 200 mm; 228 × 228 matrix; number of averages: 1/2/5/9 at b-values of 0/50/400/1,000 s/mm2; b-values of 1,400 s/mm2 were calculated from acquired lower b-values), axial dynamic contrast-enhanced (DCE) images (3D gradient echo volume-interpolated sequence; slice thickness: 3 mm; no gap; TR: 4.09 ms; TE: 1.83 ms; flip angle: 12 °; field-of-view: 260 × 260 mm; 224 × 224 matrix; temporal resolution: < 5 s). Until June 2017, diffusion-weighted parameters were slightly different affecting 12 patients (TR: 4000 ms; TE: 72 ms; number of averages: 2/5/9 at b-values of 50/400/800 s/mm2; b-values of 1,400 s/mm2 were calculated from acquired lower b-values). No endorectal coils were used. Before scanning intravenous administration of body weight adapted Butylscopolamine was administered. Axial T2 weighted images were then processed for MRI-TRUS fusion biopsies by delineation of the prostate gland and detected lesions by a trained radiologist. All reports of the mpMRI examinations were generated according to the latest PI-RADS lexicon at the time during clinical work up. The reading was performed in clinical practice by various radiology residents supervised by different board-certified radiologists with variable levels of experience in prostate imaging.

### Histopathological work up

Pathological evaluation was carried out by a board-certified pathologist during clinical routine. When malignancy was suspected, another board-certified pathologist confirmed the diagnosis. Biopsies were analyzed according to national guidelines, Tumor cell content and grading according to Gleason was evaluated [12].

### Transperineal MRI-US fusion-biopsy

Biopsy sampling was carried out via a MRI-guided transperineal ultrasound fusion technique in the MonaLisa environment (Biobot Surgical Pte Ltd, Singapore). A 3D model of the prostate gland was derived from ultrasound and co-registered to the MRI segmentations. The software’s algorithm accounted for potential deviations between the MRI-derived and ultrasound-derived model. A trained urologist performed the biopsy via two perineal incisions in a sterile setting in an operating room. Targeted biopsies were performed prior to systematic biopsies according to the volume adapted Ginsburg scheme [13].

### AI algorithm

The study employed an AI-driven commercial software for the automatic detection, segmentation, and classification of prostate lesions based on biparametric MRI (*syngo*.via MR Prostate, VB60S HF01, Siemens Healthineers). The AI model is described in detail in the appendix S1. The process entails pre-processing through automatic segmentation of the prostate gland, followed by co-registration of T2w and DWI. A 2D image-to-image convolutional neural network (CNN) produces an initial map of suspicious lesions, which are then processed by a second CNN, performing a 3D patch-wise classification for false positive reduction [14–17]. Finally, a PI-RADS score is deduced from the network’s response (Level of Suspicion, LoS) and the lesion diameter: LoS < 60: PI-RADS ≤ 2; LoS 60–79: PI-RADS 3; LoS ≥ 80: PI-RADS 4 if maximum lesion diameter < 1.5 cm, otherwise PI-RADS 5. Suspected clinically relevant lesions (PI-RADS ≥ 3) are automatically detected. For PI-RADS ≤ 2 no lesion is shown.

### Definitions and criteria

sPCa was defined based on histopathology-defined ISUP grade ≥ 2 [12].

### Data collection and analysis

The cohort was derived from our local database. The PI-RADS scores and localizations of all lesions that had been verified by targeted biopsy were recorded from the radiology reports. A radiologist (A.N.) blinded to the reports and clinical information evaluated whether the AI algorithm correctly identified the originally described lesion (overlap of 25% considered a positive match), recorded the algorithm's PI-RADS scores, and noted if the algorithm detected additional lesions. ROC analysis was performed for the original reading and the AI algorithm on a lesion level and patient level (lesion level AI algorithm only for lesions with histopathological verification; on patient level, the maximum ISUP grade detected in targeted and systematic biopsy was considered as the reference standard). ROC curves were compared with the method developed by DeLong [18]. Weighted Cohen’s Kappa was used to compare PI-RADS categories on lesion and patient level for radiology reports and AI results. Cross tables of PI-RADS categories were compared using Fisher’s exact test [19]. Data analysis was performed with R version 4.3.2 [20]. A p-value of ≤ 0.05 was considered statistically significant.

## Results

### Study population demographics

Among 272 patients the median age was 66 years (mean = 65.5, SD = 7.8 years, IQR = 12 years). Mean PSA prior to biopsy was 11.1 ng/ml (SD = 8.2 ng/ml). Mean PSA level of patients with sPCa (ISUP ≥ 2) was 12.3 ng/ml (SD = 9.4 ng/ml), mean PSA level of patients without sPCa was 9.8 ng/ml (SD = 6.6 ng/ml). Mean prostate volume segmented by the AI algorithm was 57 ml (range = 9–208 ml, SD = 33 ml).

### Biopsy

The analysis of 272 biopsies resulted in a total of 436 histopathologically verified target lesions. The mean number of biopsy cores was 34.4 including systematic biopsy and target lesion biopsy (SD = 5.7, range = 16–54, median = 34). The mean number of biopsy cores per target lesion was 2.7 (SD = 1.1, range = 1–9, median = 3).

### Patient level analysis

135/272 (49.6%) patients had clinically significant prostate cancer (sPCa, ISUP ≥ 2), 35/272 (12.9%) had clinically insignificant PCa (iPCa, ISUP = 1) and 102/272 (37.5%) had no PCa. 62/261 (23.8%) patients rated ≥ PI-RADS 3 in human reading were correctly classified as true negative by the AI, of which 13 had iPCa. 8/261 (3.1%) patients with sPCa rated ≥ PI-RADS 3 in human reading were classified false negative by the AI of which only 4 had sPCa in the targeted biopsy.

**Fig. 1:**
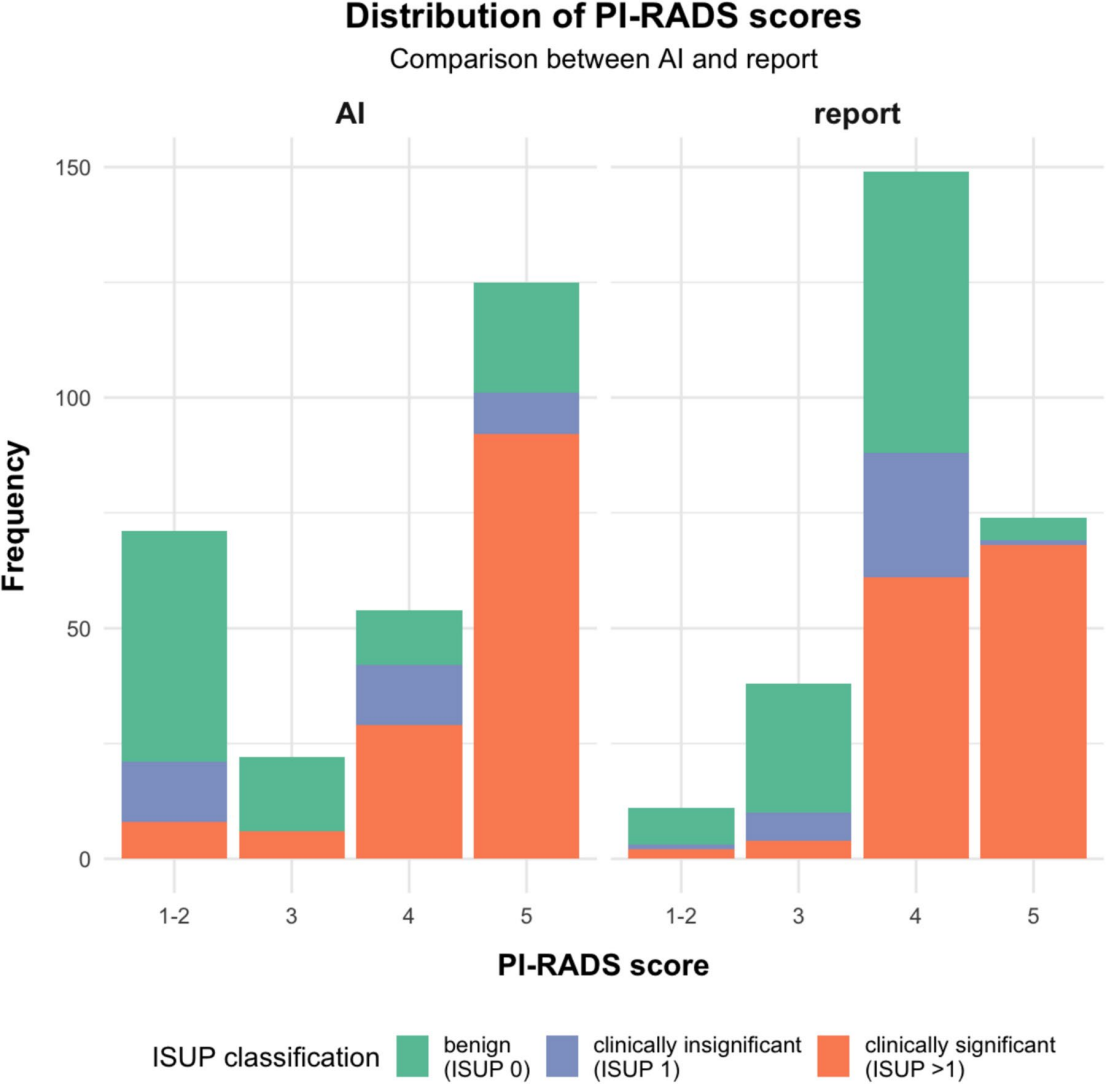
Distribution of PI-RADS scores on patient level. The filling of the bars reveals the proportion of clinically significant PCa by showing the ISUP classification of the TRUS fusion biopsy (green: benign (ISUP 0), blue: clinically insignificant (ISUP = 1), orange: clinically significant (ISUP > 1))

Distribution of PI-RADS scores for radiology reports and the AI algorithm on patient level are shown in Table 1 and Fig. 1, the corresponding confusion matrix is depicted in Table 2. Cancer detection rates of sPCa differed significantly between the AI algorithm and the radiological reports for PI-RADS 5 (p-value < 0.01), but not for other PI-RADS scores (PI-RADS 4, 3 and 1–2: p-value = 0.11, 0.15 and 0.62, respectively).

**Table 1:** Patient characteristics and distribution of the PI-RADS scores on patient level

Patient level analysis (n = 272)			
	ISUP 0 (n = 102)	ISUP 1 (n = 35)	ISUP > 1 (n = 135)
Age	62 (57, 67)^1^	66 (61, 70)^1^	68 (63, 74)^1^
PSA	8 (6, 11)^1^	8 (5, 12)^1^	10 (7, 15)^1^
PI-RADS score AI			
1–2	50/71 (70%)	13/71 (18%)	8/71 (**11%**)
3	16/22 (73%)	0/22 (0%)	6/22 (**27%**)
4	12/54 (22%)	13/54 (24%)	29/54 (**54%**)
5	24/125 (19%)	9/125 (7.2%)	92/125 (**74%**)
PI-RADS score report			
1–2	8/11 (73%)	1/11 (9.1%)	2/11 (**18%**)
3	28/38 (74%)	6/38 (16%)	4/38 (**11%**)
4	61/149 (41%)	27/149 (18%)	61/149 (**41%**)
5	5/74 (6.8%)	1/74 (1.4%)	68/74 (**92%**)

^1^Median (IQR); n (%). Cancer detection rates for clinically significant prostate cancer are marked bold. IQR: Interquartile range; ISUP: International society of urological pathology; PI-RADS: Prostate imaging reporting and data system; PSA: Prostate specific antigen

**Table 2:** Confusion matrix of the distribution of PI-RADS scores by the AI algorithm and the radiological reports on patient level

Confusion Matrix of AI vs. Human PI-RADS Scores				
	Radiological reports PI-RADS			
AI PI-RADS	1–2	3	4	5
1–2	1	21	48	1
3	2	7	11	2
4	5	4	41	4
5	3	6	49	67

**Fig. 2:**
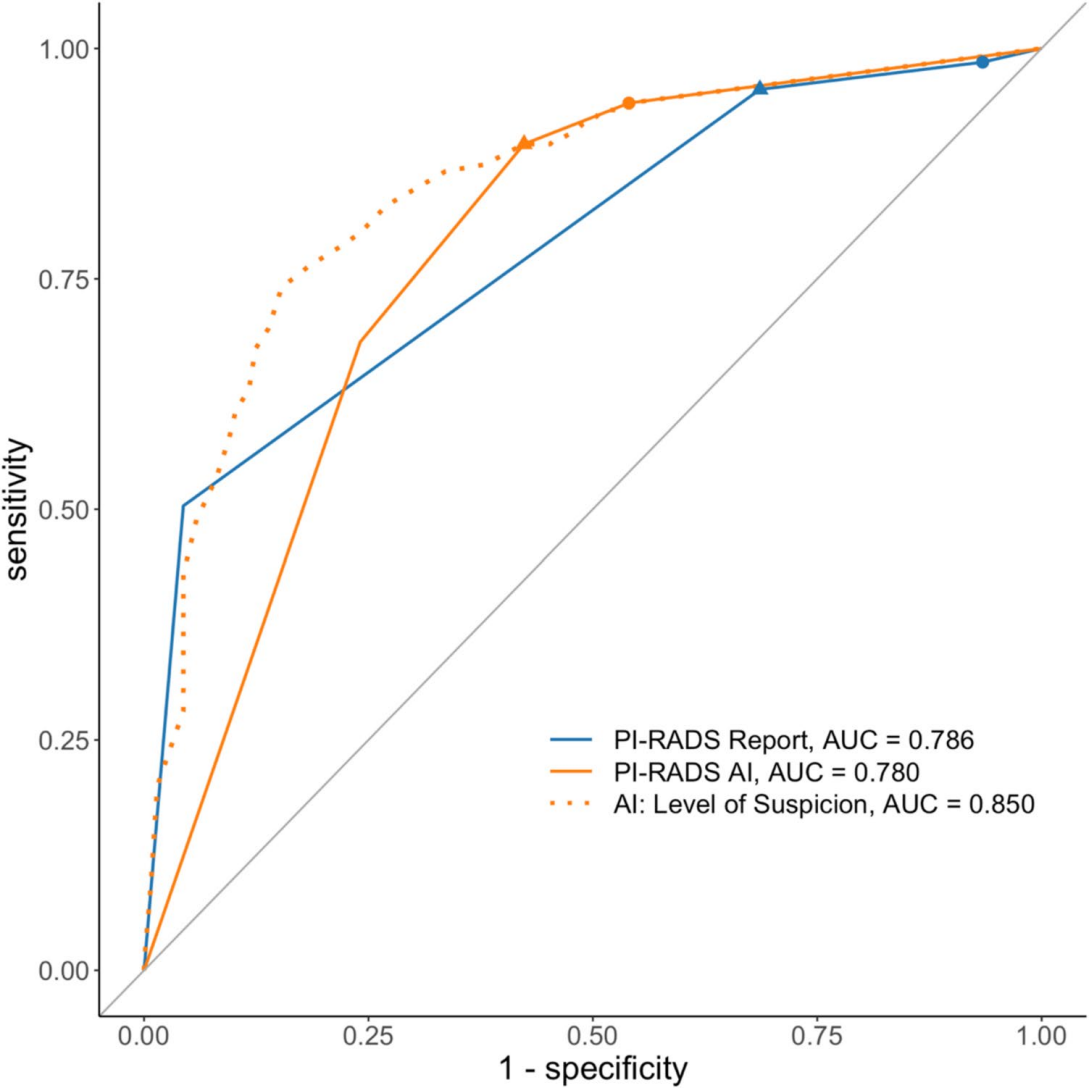
ROC analysis of AI and human reporting. The ROC curves of the AI showed higher specificities at high sensitivities compared to the ROC curve of the radiological reports. Thresholds for the PI-RADS categories 3 and 4 are indicated by triangles (PI-RADS 4) and circles (PI-RADS 3). AUC: Area under the curve; PI-RADS: Prostate imaging reporting and data system

ROC analysis with sPCa as outcome resulted in an AUC value of 0.786 for the radiological reports, 0.780 for the AI algorithm with PI-RADS classification, and 0.850 for the interim result of the AI algorithm, the “Level of Suspicion” (LoS) of each lesion, which in a subsequent step determines the PIRADS category depending on the lesion diameter (see methods). The AUC value of the LoS was significantly higher than that of the radiology reports (p < 0.05) and the PI-RADS classification of the AI algorithm (p < 0.01). There was no statistically significant difference between the AUC value of the radiology reports and the PI-RADS classification of the AI algorithm (p = 0.83). However, at a patient-level sensitivity of 90%, false-positive detection rate differed significantly with 61% (95% CI: 52–69%) for the radiological reports and 44% (95% CI: 34–57%) for the PI-RADS classification of the AI algorithm (p < 0.01). Compare to Fig. 2 for detailed information.

Radiological reports had a sensitivity of 95.6% and a specificity of 31.4% (threshold PI-RADS ≥ 4), resulting in an overall accuracy of 63.2% (95% CI: 57.2%-69.0%) for the detection of sPCa. In comparison, AI had a sensitivity of 89.6% and a specificity of 57.7%, leading to an overall accuracy of 73.5% (95% CI: 67.9%-78.7%). The positive predictive value (PPV) was 57.9% for the radiology reports and 67.6% for the AI, while the negative predictive value (NPV) was 87.8% for the radiology reports and 85.0% for the AI.

At a threshold of PI-RADS ≥ 3, radiological reports had a sensitivity of 98.5% and a specificity of 6.6% with an overall accuracy of 52.2% (95% CI: 46.1%-58.3%) for the detection of sPCa whereas AI had a sensitivity of 94.1% and a specificity of 46.0% with an overall accuracy of 70.0% (95% CI: 64.0%-75.3%). PPV was 51.0% for the radiology reports and 63.2% for the AI, NPV was 81.8% for the radiology reports and 88.7% for the AI.

Distribution of PI-RADS scores assigned by AI algorithm and radiological reports showed a fair agreement with a weighted Cohen’s Kappa of 0.38 (p < 0.001).

### Lesion level analysis

**Fig. 3:**
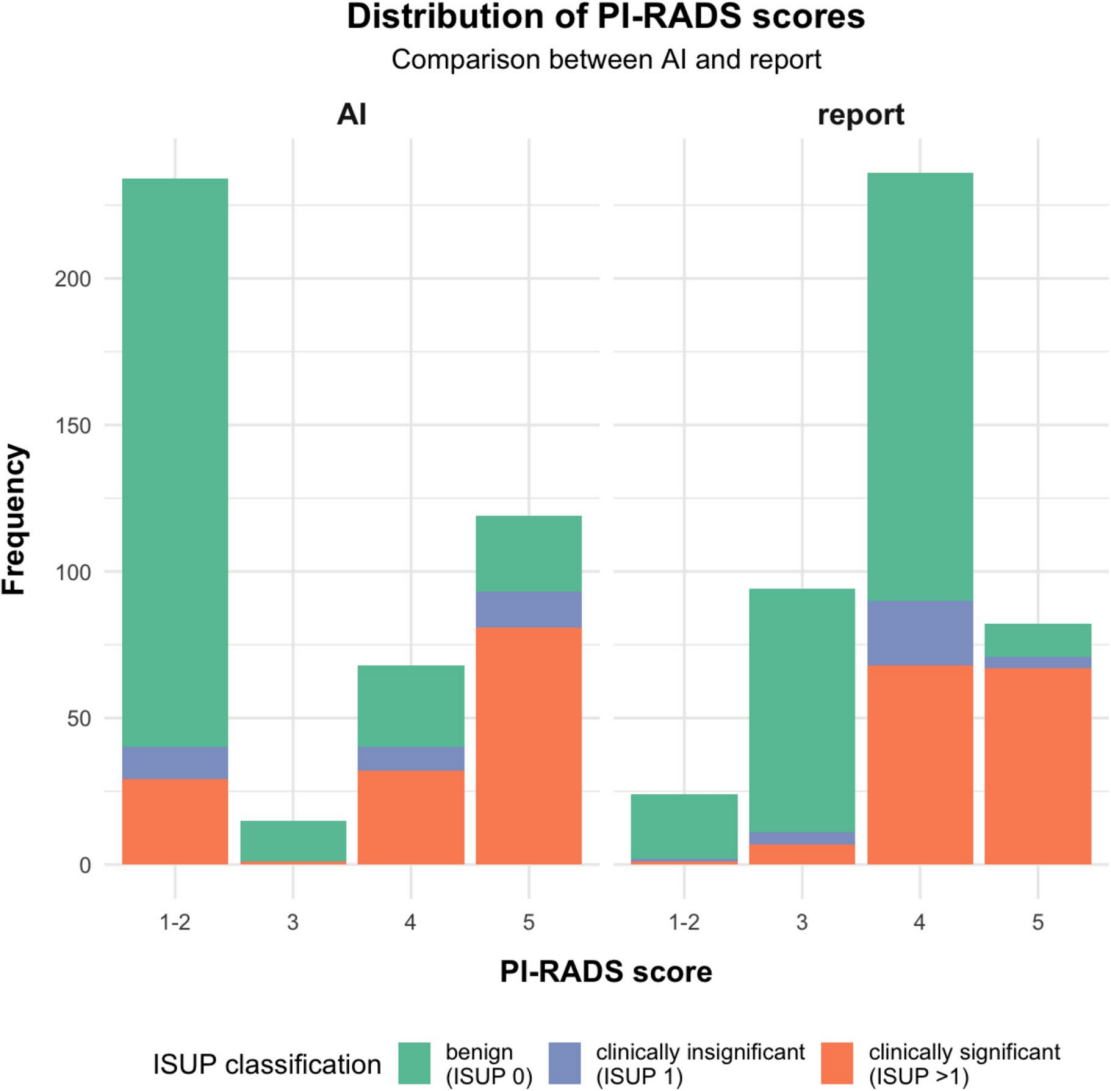
Distribution of PI-RADS scores on lesion level. The filling of the bars reveals the proportion of clinically significant PCa by showing the ISUP classification of the TRUS fusion biopsy (green: benign (ISUP 0), blue: clinically insignificant (ISUP = 1), orange: clinically significant (ISUP > 1))

Both the distribution of PI-RADS scores for radiology reports and AI and the CDR on lesion level are shown in Table 3 and Fig. 3. CDR of sPCa differed significantly between AI and radiological reports for PI-RADS 4 (p-value < 0.01) and PI-RADS 5 (p-value < 0.05), but not for the other PI-RADS scores (PI-RADS 3: p-value = 1 and PI-RADS 1–2: p-value = 0.33).

**Table 3:** Patient characteristics and distribution of the PI-RADS scores on lesion level

Lesion level analysis (n = 436)			
	ISUP 0 (n = 262)	ISUP 1 (n = 31)	ISUP > 1 (n = 143)
Age	63 (58, 68)^1^	67 (63, 71)^1^	70 (66, 76)^1^
PSA	8 (6, 12)^1^	8 (7, 10)^1^	11 (7, 16)^1^
PI-RADS score AI			
1–2	194/234 (83%)	11/234 (4.7%)	29/234 (**12%**)
3	14/15 (93%)	0/15 (0%)	1/15 (**6.7%**)
4	28/68 (41%)	8/68 (12%)	32/68 (**47%**)
5	26/119 (22%)	12/119 (10%)	81/119 (**68%**)
PI-RADS score report			
1–2	22/24 (92%)	1/24 (4.2%)	1/24 (**4.2%**)
3	83/94 (88%)	4/94 (4.3%)	7/94 (**7.4%**)
4	146/236 (62%)	22/236 (9.3%)	68/236 (**29%**)
5	11/82 (13%)	4/82 (4.9%)	67/82 (**82%**)

^1^Median (IQR); n (%). Cancer detection rates for clinically significant prostate cancer are marked bold. IQR: Interquartile range; ISUP: International society of urological pathology; PI-RADS: Prostate imaging reporting and data system; PSA: Prostate specific antigen

342 target lesions were located in the peripheral zone (PZ), revealing 112 (32.7%) sPCa, 26 (7.6%) iPCa, and 204 (59.6%) benign lesions. 79 were located in the transition zone (TZ), revealing 19 (24.1%) sPCa, 5 (6.3%) iPCa, and 55 (69.6%) benign lesions. 15 target lesions were located in both zones, revealing 12 (80%) sPCa, 0 (0%) iPCa, and 3 (20%) benign lesions (although sPCa was found in the systematic biopsies in all cases). The distribution of the PI-RADS scores for the radiology reports and for the AI and cancer detection rates on lesion level stratified by zones are shown in Table 4. AUC was 0.77 vs. 0.75 in the PZ, 0.83 vs 0.81 in the TZ and 0.67 vs. 0.79 in overlapping lesions for AI and human reading, respectively.

**Table 4:** Patient characteristics and distribution of the PI-RADS scores on lesion level stratified by zones

a) Peripheral zone—lesion level analysis (n = 342)			
	ISUP 0 (n = 204)	ISUP 1 (n = 26)	ISUP > 1 (n = 112)
Age	62 (58, 68)^1^	67 (63, 71)^1^	69 (66, 76)^1^
PSA	8.0 (5.6, 11.4)^1^	8.7 (7.0, 10.5)^1^	11.0 (6.5, 16.0)^1^
PI-RADS score AI			
1–2	151/188 (80%)	11/188 (5.9%)	26/188 (**14%**)
3	11/12 (92%)	0/12 (0%)	1/12 (**8.3%**)
4	23/57 (40%)	5/57 (8.8%)	29/57 (**51%**)
5	19/85 (22%)	10/85 (12%)	56/85 (**66%**)
PI-RADS score report			
1–2	15/17 (88%)	1/17 (5.9%)	1/17 (**5.9%**)
3	47/51 (92%)	2/51 (3.9%)	2/51 (**3.9%**)
4	133/215 (62%)	20/215 (9.3%)	62/215 (**29%**)
5	9/59 (15%)	3/59 (5.1%)	47/59 (**80%**)

b) Transition zone—lesion level analysis (n = 79)			
	ISUP 0 (n = 55)	ISUP 1 (n = 5)	ISUP > 1 (n = 19)
Age	63 (58, 68)^1^	66 (61, 68)^1^	71 (67, 75)^1^
PSA	10 (6, 17)^1^	8 (7, 9)^1^	10 (9, 13)^1^
PI-RADS score AI			
1–2	42/45 (93%)	0/45 (0%)	3/45 (**6.7%**)
3	3/3 (100%)	0/3 (0%)	0/3 (**0%**)
4	5/11 (45%)	3/11 (27%)	3/11 (**27%**)
5	5/20 (25%)	2/20 (10%)	13/20 (**65%**)
PI-RADS score report			
1–2	7/7 (100%)	0/7 (0%)	0/7 (**0%**)
3	36/43 (84%)	2/43 (4.7%)	5/43 (**12%**)
4	11/18 (61%)	2/18 (11%)	5/18 (**28%**)
5	1/11 (9.1%)	1/11 (9.1%)	9/11 (**82%**)

c) Crossing zones—lesion level analysis (n = 15)		
	ISUP 0 (n = 3)	ISUP > 1 (n = 12)
Age	69 (68, 71)^1^	70 (65, 76)^1^
PSA	9 (8, 12)^1^	14 (8, 18)^1^
PI-RADS score AI		
1–2	1/1 (100%)	0/1 (**0%**)
5	2/14 (14%)	12/14 (**86%**)
PI-RADS score report		
4	2/3 (67%)	1/3 (**33%**)
5	1/12 (8.3%)	11/12 (**92%**)

(a) peripheral zone; (b) transition zone; (c) crossing zone; all patients with lesions that grew across zones had sPCa in the systematic biopsy

^1^Median (IQR); n (%). Cancer detection rates for clinically significant prostate cancer are marked bold. IQR: Interquartile range; ISUP: International society of urological pathology; PI-RADS: Prostate imaging reporting and data system; PSA: Prostate specific antigen

At a threshold of PI-RADS ≥ 4, radiological reports had a sensitivity of 94.4% and a specificity of 37.5% with an overall accuracy of 56.2% (95% CI: 51.4%-60.9%) for the detection of sPCa whereas AI had a sensitivity of 79.0% and a specificity of 74.7% with an overall accuracy of 76.2% (95% CI: 71.9%-80.0%). In this setting, the PPV was 42.5% for radiology reports and 60.4% for the AI algorithm, while NPV was 93.2% for the radiology reports and 88.0% for AI.

At a threshold of PI-RADS ≥ 3, radiological reports had a sensitivity of 99.3% and a specificity of 7.9% with an overall accuracy of 37.8% (95% CI: 33.3%-42.6%) for the detection of sPCa whereas AI had a sensitivity of 79.7% and a specificity of 70.0% with an overall accuracy of 73.2% (95% CI: 68.7%-77.3%). The PPV was 34.4% for radiology reports and 56.4% for AI, the NPV was 95.8% for radiology reports and 87.6% for AI.

**Fig. 4:**
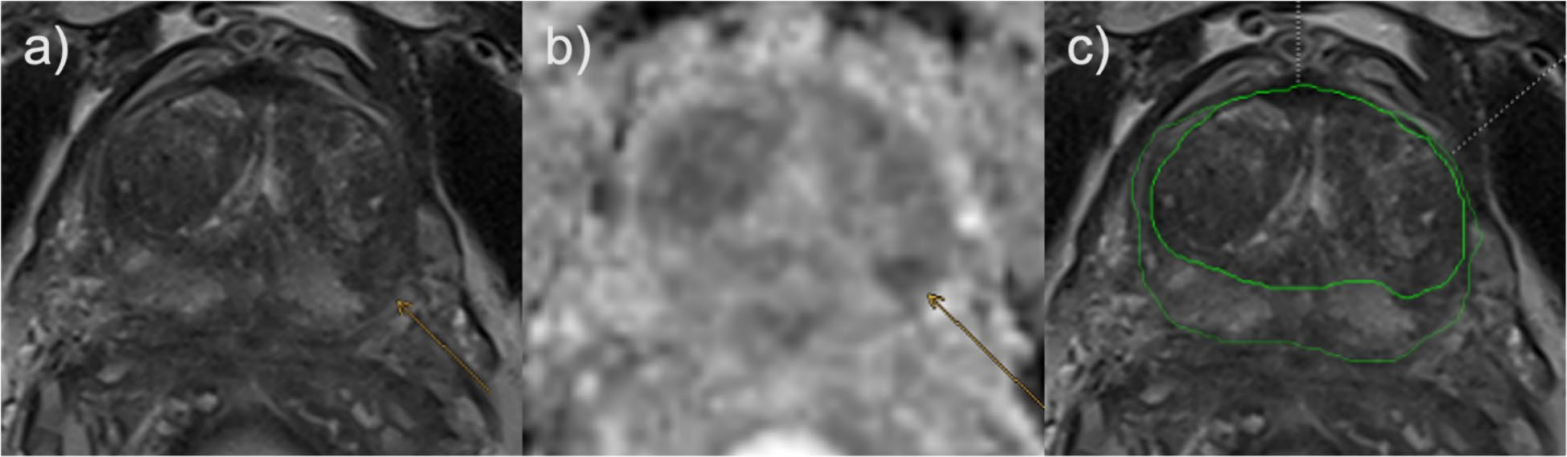
False negative example regarding AI reading; lesion not detected by AI. Suspected lesion in the left posterolateral peripheral zone. 77 years old patient with a PSA value of 10.9 ng/ml and no prior biopsy. Radiologists assigned a PI-RADS score of 4, AI did not detect the lesion. Targeted biopsy revealed Gleason 4 + 3 (ISUP 3) in 2/3 cores, systematic biopsy yielded a maximum Gleason score of 4 + 3 in 4/22 cores (ISUP 3). Orange arrows mark the suspected lesion. **a** axial T2 TSE; **b** axial ADC; **c** axial T2 TSE, green borders: AI segmentation of TZ and PZ, no lesion was detected by AI. ADC: apparent diffusion coefficient; PSA: prostate specific antigen; PZ: peripheral zone; TSE: turbo spin echo; TZ: transitional zone

**Fig. 5:**
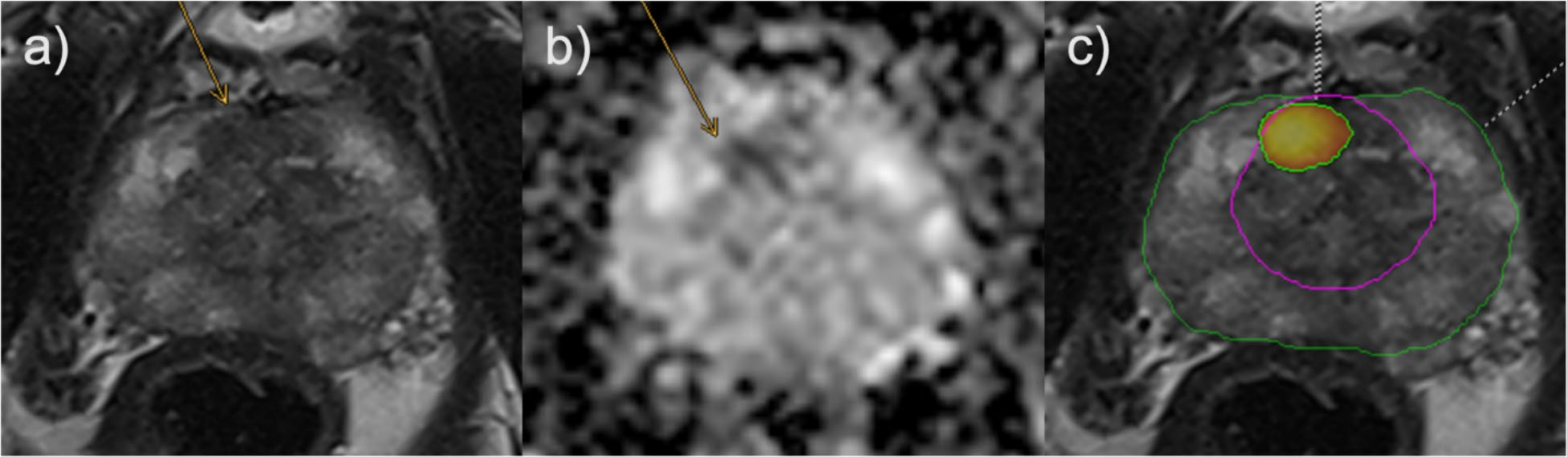
False negative example regarding human reading; lesion detected but falsely classified by radiologist, detected and classified correctly by AI. Suspected lesion in the anterior right transitional zone. 66 years old patient with a PSA value of 6.26 ng/ml and no prior biopsy. Radiologists assigned a PI-RADS score of 2, AI assigned a score of 4. Targeted biopsy revealed Gleason 4 + 3 in 2/3 cores (ISUP 3) and Gleason 4 + 4 in 1/3 cores (ISUP 4), systematic biopsy yielded a maximum Gleason score of 3 + 3 in 1/23 cores (ISUP 1). Orange arrows mark the suspected lesion. **a** axial T2 TSE; **b** axial ADC; **c** axial T2 TSE, green border: AI segmentation of PZ, purple border: AI segmentation of TZ. ADC: apparent diffusion coefficient; PSA: prostate specific antigen; PZ: peripheral zone; TSE: turbo spin echo; TZ: transitional zone

**Fig. 6:**
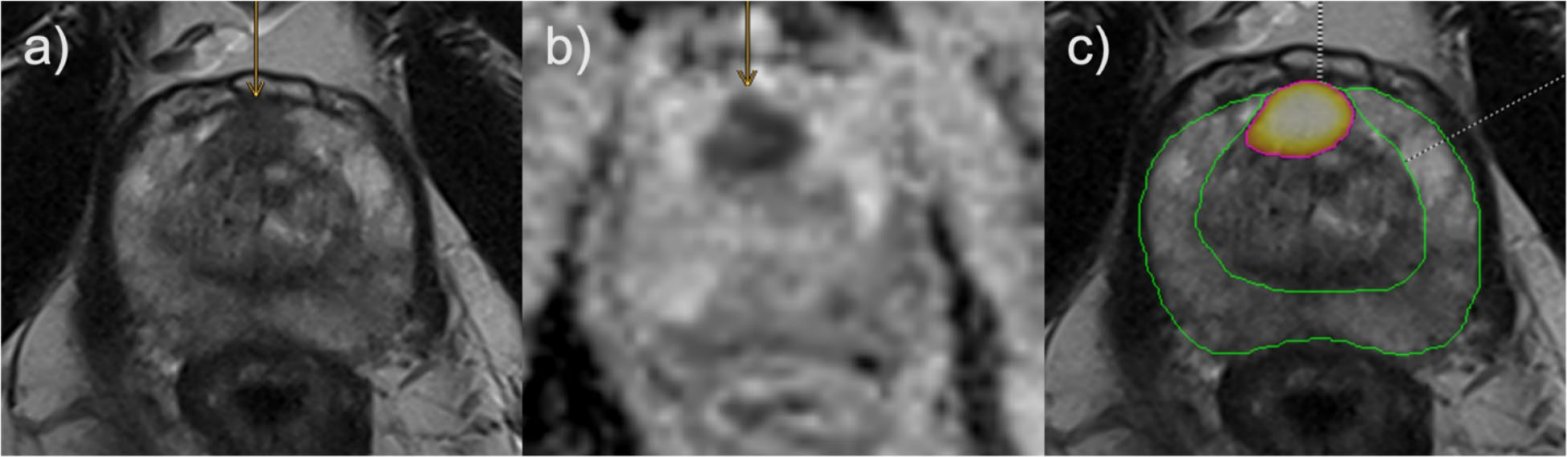
True positive example regarding AI reading; lesion detected and classified correctly by AI. Suspected lesion in both the anterior peripheral and transitional zone. 65 years old patient with a PSA value of 9.22 ng/ml and no prior biopsy. Radiologists assigned a PI-RADS score of 4, AI assigned a score of 4. Targeted biopsy revealed Gleason 3 + 4 in 1/2 cores (ISUP 2), systematic biopsy yielded a maximum Gleason score of 3 + 4 in 1/32 cores (ISUP 2). Orange arrows mark the suspected lesion. **a** axial T2 TSE; **b** axial ADC; **c** axial T2 TSE, green borders: AI segmentation of TZ and PZ. ADC: Apparent diffusion coefficient; PSA: Prostate specific antigen; PZ: Peripheral zone; TSE: Turbo spin echo; TZ: Transitional zone

Figures 4, 5, 6 depict exemplary cases of false negative AI reading, false negative human reading with correct AI classification and true positive AI reading.

## Discussion

AI reading showed robust results in detecting sPCa compared to human reports on both patient and lesion level. At a threshold of PI-RADS ≥ 4, sensitivities derived from the AI were slightly lower than those of human reading (AI vs. radiologists: 89.6% vs. 95.6% on patient level, 79.0% vs. 94.4% on lesion level). However, AI outperformed human reading in specificity (57.7% vs. 31.4%, 74.7% vs. 37.5%), overall diagnostic accuracy (73.5% vs. 63.2%, 76.2% vs. 56.2%) and PPV (67.6% vs. 57.9%, 60.4% vs. 42.5%). NPV of the AI was slightly lower compared to human reading (85.0% vs. 87.8%, 88.0% vs. 93.2%).

At a threshold of PI-RADS ≥ 3, sensitivities derived from the AI were still lower than those derived from human reading (94.1% vs. 98.5% on patient level; 79.7% vs. 99.3% on lesion level). AI still outperformed human reading in specificity (46.0% vs. 6.6%, 70.0% vs. 7.9%), overall diagnostic accuracy (70.0% vs. 52.2%, 73.2% vs. 37.8%) and PPV (63.2% vs. 51.0%, 56.4% vs. 34.4%). NPV of the AI was higher on patient level (AI: 88.7%; radiologists: 81.8%), but slightly lower on lesion level (AI: 87.6%; radiologists: 95.8%).

These results are partially in line with recent findings of a large multi-center study by Saha et al. where biparametric AI detection outperformed human evaluation in re-reading of a sample of their subcohort by experienced uroradiologists (AUC of AI: 0.91 vs. AUC of radiologists: 0.86), detecting more sPCa at the same specificity (57.7%) as well as reducing false positive rates and decreasing detection of insignificant PCa at a sensitivity of 89.4%) [21]. Compared to their results, we found equally high sensitivities for both AI and human reading but substantially lower specificities. Furthermore, the ROC analysis resulted in lower AUC values for both human and AI reports (0.786 for radiological reports and 0.780 for AI). However, when focusing on the high-sensitivity region of the ROC curve, the AI was superior to the radiological reports and showed significantly higher specificities, resulting in lower false positive rates, which is desirable when MRI is used as a triage tool. Notably, Penzkofer et al. recommend a sensitivity of at least 90% [22]. Moreover, our findings generally fit within the ranges reported by a recent systematic review (AI: AUC 0.78–0.95, sensitivity 57–98.6%, specificity 35–85.8%; radiologists: AUC 0.80–0.92, sensitivity 59–100%, specificity 6–100%) [23].

Deviations in sensitivity of human and AI reading may be due to lacking implementation of clinical information into the algorithm which holds an advantage for human readers. The sensitivity in human reading was comparable to and its specificity lower than findings in large-scale studies on patient level for a threshold of PI-RADS ≥ 4 (sensitivity/specificity: 95.6%/31.4%). Specificity was exceptionally low for a threshold of PI-RADS ≥ 3 (sensitivity/specificity: 98.5%/6.6%). Current estimates range around a sensitivity/specificity of 89%/66% for a threshold of PI-RADS ≥ 4 and 96%/43% for a threshold of PI-RADS ≥ 3 regarding sPCa, although acknowledging wide ranges and heterogenous results for specificity [24, 25]. Pooled sensitivity/specificity is currently estimated at around 87%/74% [26]. AI reporting in our study matched or outperformed these estimates while specificity in human reporting was substantially lower.

Low specificities in human reading are likely attributable to differences in the underlying patient cohorts, as examinations with PI-RADS scores of ≤ 2 are underrepresented due to the frequent avoidance of biopsy in such cases. Additionally, the setting in the clinical routine where exams are first read by a resident and then supervised by a board-certified radiologist might lead to over-reporting of PI-RADS ≥ 4 lesions resulting in higher sensitivities and lower specificities because of the tendency to classify indeterminate lesions as malignant to avoid missing significant cancer. Furthermore, the fact that mpMRI readings were not exclusively performed by uroradiologists might have also contributed to the tendency to identify indeterminate lesions as potentially malignant, leading to over-reporting of insignificant PCa and benign findings.

CDR for sPCa on patient level in our study were 11% for PI-RADS ≤ 2, 27% for PI-RADS 3, 54% for PI-RADS 4, 74% for PI-RADS 5 in AI reading and 18% for PI-RADS ≤ 2, 11% for PI-RADS 3, 41% for PI-RADS 4 and 92% for PI-RADS 5 in human reading, respectively. This differs partially from recent findings from a meta-analysis that showed detection rates of 5–6% in PI-RADS ≤ 2, 19% in PI-RADS 3, 54% in PI-RADS 4, 84% in PI-RADS 5 but might be due to the limited sample size of this analysis [25]. Furthermore, the distribution of PI-RADS scores and their corresponding Gleason score in biopsy in Table 1 shows outliers and different rating patterns between AI and human reading. On one hand AI allocated more PI-RADS 1 and 2 correctly to benign findings but also shows a spike in falsely allocated PI-RADS 5 scores on the other hand, which cannot be explained sufficiently by the current data. Interestingly, human reading shows a cluster of PI-RADS 3 and 4 scores in the ISUP 0 category which might stem from ambiguity of lesions in imaging and the tendency to secure the diagnosis by biopsy.

Limitations of our study are mainly its retrospective and monocentric design. Furthermore, the cohort shows a slight overrepresentation of GG > 1 PCa subtypes, which implies that the study might be prone to selection bias, as referrals to university care and region-specific ethnicity may result in a filtered patient cohort that differs from other study collectives. This may also be a consequence of the extensive systematic biopsies conducted at our institution. Furthermore, the study design was based on patients with histopathological confirmation. This means that the vast majority of patients had positive MRI findings on human reading and that negative MRI scans are under-represented, resulting in a cohort that is not fully representative of the broader patient population. This selection bias may also explain the high sensitivity observed in the radiology reports. Lastly, the cohort size of 272 patients was insufficient to allow for robust stratification by PSA levels, age, or lesion subgroups. Future prospective, multi-center trials with larger populations are necessary to confirm and expand upon our observations.

## Conclusion

The AI-based algorithm proved to be reliable and robust for the detection of sPCa and PI-RADS scoring. The CDRs and distribution of PI-RADS assessment categories of the AI algorithm are consistent with the results of current meta-analyses, indicating precise risk stratification. In addition, the AI algorithm correctly classified 24% of patients scored as PI-RADS ≥ 3 in human reading as true negatives for sPCa, potentially reducing unnecessary biopsies. Moreover, it missed only 3% of the sPCa cases in this group, with only 1.5% being detected in the targeted biopsy. In anticipation of further validation in large multi-center cohorts, the algorithm seems suitable for implementation in routine clinical workflows alongside human reading, potentially increasing efficiency and providing reader reassurance.

## Supplementary Information

Below is the link to the electronic supplementary material.

Supplementary file1 (DOCX 30 KB)
