# Supplementary material for: Diagnostic performance of a fully automated AI algorithm for lesion detection and PI-RADS classification in patients with suspected prostate cancer
Source: Radiol Med. 2025 Apr 17;130(7):1039–49. doi: 10.1007/s11547-025-02003-0 (PMC12263473; doi:10.1007/s11547-025-02003-0)
Supplement: Supplementary file 1 — Supplementary file1 (DOCX 30 KB) [file 11547_2025_2003_MOESM1_ESM.docx]

## APPENDIX S1: DETAILED DESCRIPTION OF THE PROSTATE AI ALGORITHM

### Overview

In this study, a commercially available deep learning–based computer-aided diagnosis (DL-CAD) system (*syngo*.via MR Prostate, VB60S HF01, Siemens Healthineers) was used for the detection and classification of prostate lesions based on biparametric MRI (bp-MRI) examinations. A prior version of this DL-CAD system was described earlier [14]. It is composed of a preprocessing pipeline and a deep learning–based lesion detection and classification algorithm, using a database of 3,087 bp-MRI examinations from multiple institutions for training and testing. The overall processing steps can be described as follows: the bp-MRI data are first parsed to select T2-weighted (T2w) and diffusion-weighted (DWI) acquisitions; then, an apparent diffusion coefficient (ADC) map and a synthetic DWI-b2000 image are calculated. After that, a deep learning–based whole-prostate segmentation is conducted on the T2w images and diffusion-weighted images, followed by rigid registration and resampling of the DWI-b2000 and ADC images to the T2w reference frame.

The subsequent deep learning-based processing stages first determine lesion candidates, and then apply a second step to reduce false-positive detections, resulting in an AI level of suspicion (LoS) score for each candidate. Based on this, the lesion candidates are either rejected as Prostate Imaging Reporting and Data System (PI-RADS) 1-2 or classified as PI-RADS 3, 4, or 5.

The output of the AI algorithm contains a suspicion map indicating the detection network’s voxel-wise response. For each detected lesion, the LoS, the PI-RADS score and a three-dimensional lesion contour are provided. Segmentation masks of the prostate gland, peripheral zone, and non-peripheral zone are also included for anatomic localization.

### Preprocessing Pipeline

In the preprocessing stage, the pipeline searches incoming DICOM files and extracts axial T2w and acquired DWI series. From the DWI series, the ADC map and a synthetic DWI-b2000 image is calculated. A deep learning method, similar to the adversarial network introduced by Yang *et al.* [15] is employed to segment the prostate gland in the T2w volume and the DWI. This approach leverages a convolutional encoder–decoder framework and can handle anatomical variations and fuzzy organ boundaries. Following prostate segmentation, a rigid registration based on a projection-based method described by Khamene *et al.* [16] is used to align the DWI-b2000 and ADC images to the T2w sequence for consistent anatomical localization.

### Lesion Detection

After preprocessing, the detection network automatically identifies suspicious areas within the prostate that could represent clinically significant lesions (PI-RADS ≥ 3) using a 2D convolutional image-to-image network. The detection network has been trained to respond strongly to abnormal signals in T2w, ADC, and DWI-b2000 images.

### Lesion Classification

In the second stage of the pipeline, each automatically detected lesion is evaluated by a deep learning–based classification model that refines the lesion’s category and reduces false positives. This approach follows the design principles described by Yu *et al.* [17]. The classification model employs multiscale images and contextual information from T2w, ADC, and DWI-b2000 acquisitions, thereby improving specificity, and has been trained considering the PI-RADS scores but also corresponding results from lesion-targeted biopsy as ground truth labels. Each lesion is assigned a LoS score ranging from 60-100, indicating the likelihood of being a significant lesion. The final PI-RADS score is derived from the LoS and the lesion’s diameter: LoS < 60: PI-RADS ≤ 2 (lesion candidate is rejected); LoS 60-79: PI-RADS 3; LoS ≥ 80: PI-RADS 4 if maximum lesion diameter < 1.5 cm, otherwise PI-RADS 5. For PI-RADS ≤ 2, no lesion is shown.

### Training Data and Ground Truth

A collection of 3,087 bp-MRI examinations from seven institutions, mostly based on Siemens Healthineers 3 T MRI scanners, formed the training set, including 1362 lesion-free cases and 1725 cases harboring at least one lesion considered significant (PI-RADS v2.1 score of 3 or higher). In total, 2133 lesions with a PI-RADS score of 3 or higher were considered. None of these training examinations overlapped with the data used in the present work. All lesions were delineated with pixel-level annotations and assigned PI-RADS categories by radiologists. Where available, also the corresponding histopathological results from lesion-targeted biopsy were utilized in the lesion classification step. Thus, during training, the model learned to detect lesions marked as PI-RADS 3 or higher and to classify them accordingly, while also refining false positives through additional contextual features. The techniques of data augmentation, such as in-plane rotations and translations, were employed to ensure robustness to variations in patient positioning, prostate shape, and MRI protocol.

### References

14. Winkel DJ, Tong A, Lou B et al (2021) A Novel Deep Learning Based Computer-Aided Diagnosis System Improves the Accuracy and Efficiency of Radiologists in Reading Biparametric Magnetic Resonance Images of the Prostate: Results of a Multireader, Multicase Study. Invest Radiol 56:605–613. https://doi.org/10.1097/RLI.0000000000000780

15. Yang D, Xu D, Zhou SK et al (2017) Automatic Liver Segmentation Using an Adversarial Image-to-Image Network. In: Descoteaux M, Maier-Hein L, Franz A, et al (eds) Medical Image Computing and Computer Assisted Intervention − MICCAI 2017. Springer International Publishing, Cham, pp 507–515

16. Khamene A, Chisu R, Wein W et al (2006) A Novel Projection Based Approach for Medical Image Registration. In: Pluim JPW, Likar B, Gerritsen FA (eds) Biomedical Image Registration. Springer Berlin Heidelberg, Berlin, Heidelberg, pp 247–256

17. Yu X, Lou B, Shi B et al (2020) False Positive Reduction Using Multiscale Contextual Features for Prostate Cancer Detection in Multi-Parametric MRI Scans. In: 2020 IEEE 17th International Symposium on Biomedical Imaging (ISBI). pp 1355–1359 https://doi.org/10.1109/ISBI45749.2020.9098338
